# Fluorometric assay for phenotypic differentiation of drug-resistant HIV mutants

**DOI:** 10.1038/srep10323

**Published:** 2015-05-19

**Authors:** Qinchang Zhu, Zhiqiang Yu, Tsutomu Kabashima, Sheng Yin, Shpend Dragusha, Ahmed F. M. El-Mahdy, Valon Ejupi, Takayuki Shibata, Masaaki Kai

**Affiliations:** 1 Faculty of Pharmaceutical Sciences, Graduate School of Biomedical Sciences, Nagasaki University, 1-14, Bunkyo-Machi, Nagasaki 852-8521, Japan

## Abstract

Convenient drug-resistance testing of viral mutants is indispensable to effective treatment of viral infection. We developed a novel fluorometric assay for phenotypic differentiation of drug-resistant mutants of human immunodeficiency virus-I protease (HIV-PR) which uses enzymatic and peptide-specific fluorescence (FL) reactions and high-performance liquid chromatography (HPLC) of three HIV-PR substrates. This assay protocol enables use of non-purified enzyme sources and multiple substrates for the enzymatic reaction. In this study, susceptibility of HIV mutations to drugs was evaluated by selective formation of three FL products after the enzymatic HIV-PR reaction. This proof-of-concept study indicates that the present HPLC-FL method could be an alternative to current phenotypic assays for the evaluation of HIV drug resistance.

Although dozens of inhibitors for human immunodeficiency virus (HIV) enzymes, such as protease (HIV-PR) and reverse transcriptase, are used to treat acquired immune deficiency syndrome (AIDS)^1^,^2^, HIV mutants with resistance to those inhibitors have been generated all over the world^3^,^4^,^5^. Thus, a facile test for HIV drug resistance is needed for the appropriate choice of inhibitors in both antiviral therapy and for prevention of mother-to-child transmissible infection^6^. Actually, HIV-resistance testing has been recommended in international HIV treatment guidelines as a standard of care for HIV-infected patients^7^,^8^. HIV-PR cleaves pro-proteins of HIV to create mature HIV virions in host cells^9^,^10^. More than 60 genetic mutations in HIV-PR indicated drug resistance in AIDS patients^11^. These mutations were located in either the drug-binding domain or distant sites in the enzyme^12^, and reduced affinity to HIV-PR inhibitors^13^,^14^,^15^,^16^.

The drug-resistant HIV mutants are currently determined by genotypic or phenotypic assays. Genotypic assays predict drug resistance based on the detection of viral genetic mutations, and are often used because of their precise evaluation and short analytical time^17^,^18^. However, novel and/or complex mutations can make accurate prediction of HIV drug resistance diffcult^19^,^20^, because unknown mutants cannot be predicted unambiguously, and their genetic information becomes more and more complicate^21^. Even well-explained drug-resistant mutations often alter phenotypic susceptibility with complex ways^22^,^23^,^24^.

On the other hand, phenotypic assays directly measure the concentration of drugs needed to inhibit HIV replication *in vitro,* and are thus more trustworthy than genotypic assays^25^,^26^. Most current phenotypic assays determine the replication of recombinant viruses containing a patient-derived HIV gene in the presence of antiviral drugs^27^,^28^,^29^. The recombinant virus is generated by the homologous recombination between a provirus vector and patient-derived genes, and cultured for approximately one week. After further titration, those viruses will be used to infect the CD4^+^ lymphocytes for the evaluation of final drug susceptibility. Such cell-based assay usually takes 3 to 4 weeks to generate results^27^,^28^,^29^,^30^, which is time-consuming and thus limits its clinical use.

Previously, we developed several simple, inexpensive and sensitive fluorescence (FL) reactions with non-FL reagents for the highly selective detection of *N*-terminal particular amino acid-containing oligopeptides. These non-FL reagents are 1) hydroxylamine, cobalt(II) and borate for *N*-terminal Tyr-containing peptides^31^, 2) glyoxal for *N*-terminal Trp-containing peptides^32^, 3) catechol (1,2-dihydroxybenzene) , NaIO_4_ and borate for N-terminal Phe-, Leu-, Val-, or Ala-containing peptides^33^, 4) catechol, NaIO_4_ and 2-[4-(2-hydroxyethyl) piperazin-1-yl] ethanesulfonic acid (HEPES) buffer (pH 7.5) for *N*-terminal Ser-containing peptides^34^, 5) 3,4-dihydroxyphenylacetic acid, NaIO_4_ and borate for *N*-terminal Gly-containing peptides^35^ and 6) 3,4-dihydroxybenzoic acid, NaIO_4_ and borate for *N*-terminal Pro-containing peptides^36^. Those reagents were applied to analyze several peptides in complex mixtures such as tissues and enzymatic digests, and also to selectively assay various enzyme activities^37^,^38^,^39^,^40^. Hence, a limited number of amino acids in peptides can be selectively converted to individual FL compounds for the sensitive assay of particular enzymes.

The present study aims to develop a high-performance liquid chromatography (HPLC)-based FL assay method that uses multiple acetyl peptide substrates for phenotypic differentiation of drug-resistant HIV-PR mutants in non-purified HIV-PR samples, utilizing the catechol, NaIO_4_ and borate reagents^33^ for the FL detection of *N*-terminal Phe-, Leu- or Val-containing peptides. Consequently, we found that the developed HFA method offers great potential for facile, informative and reliable phenotypic differentiation of HIV-PR mutations that engender drug resistance.

## Results

**Figure 1:**
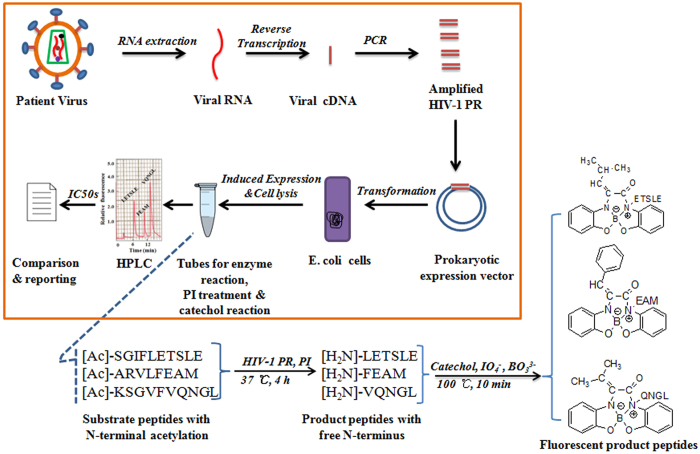
Schematic protocol of the present HPLC-based FL assay (HFA) for the differentiation of *HIV-PR* mutants. A patient-derived *HIV-PR* gene was first cloned and expressed in *E. coli* cells. Cell lysates containing HIV-PR were then directly incubated with three substrates of *N*-terminal acetyl peptides in the presence or absence of an HIV-PR inhibitor (PI). The enzymatically cleaved *N*-terminal free peptides were reacted with catechol in the presence of NaIO_4_ and borate to generate specific FL compounds, which were analyzed with HPLC.

HFA protocol for the phenotypic differentiation of drug resistant HIV-PR mutants. We developed HPLC-based FL assay (HFA) for phenotypic differentiation of drug-resistant HIV-PR mutants by using the enzymatic and peptide-specific FL reactions and HPLC analysis of three HIV-PR substrates. Figure 1 shows the schematic HFA protocol. The HIV-PR gene from an AIDS patient was first cloned into a prokaryotic expression vector and expressed in E. coli cells. The cell lysate containing HIV-PR was then directly used for enzymatic reactions with three N-terminal acetyl substrates in the absence or presence of a pharmaceutical HIV-PR inhibitor. Subsequently, the N-terminal free peptides enzymatically produced from the acetyl peptides were converted with catechol to FL compounds, which were analyzed by the following HPLC. The susceptibility of the patient’s HIV-PR was evaluated by the formation ratio of three cleaved peptides. In addition, drug resistance of HIV-PR mutants towards inhibitors was analyzed on the basis of the change of IC_50_, comparing with that of wild-type HIV-PR.

### Multiple substrates and HPLC-FL detection of enzymatic products

**Figure 2:**
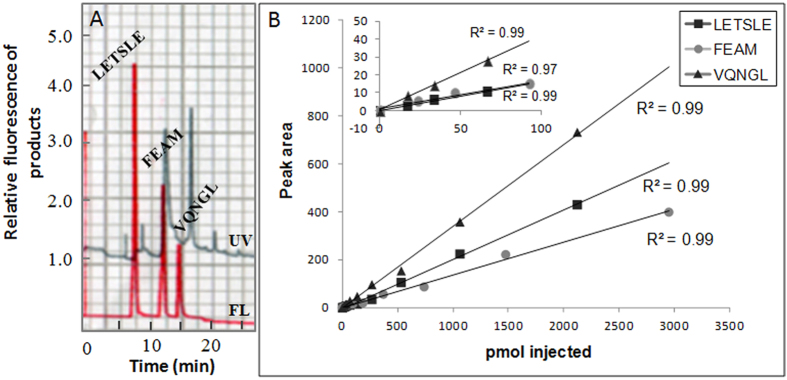
(**A**) HPLC-FL detection of three *N*-terminal free peptides of LETSLE, FEAM and VQNGL. Their peptides in a FL reaction mixture after HIV-PR enzymatic reaction were injected at 474, 1025 or 97 pmol per 20-μl injection, respectively into the reversed phase HPLC system. (**B**) Standard curves from simultaneous HPLC-FL analyses of each synthetic peptide. Peak areas are shown in arbitrary units.

Three peptide substrates, SGIFLETSLE, ARVLFEAM and KSGVFVQNGL, were selected from several HIV-PR substrates^41^,^42^,^43^,^44^,^45^ reported for wild-type HIV-PR, and used as acetyl peptides to prevent the FL reaction between substrate and catechol. To confirm suitability of the three HIV-PR substrates, their enzymatic products were reacted with catechol in the presence of NaIO_4_ and borate^33^, and then determined by reversed-phase HPLC with a fluorescence (FL) detector (Fig. 2).

Three FL peaks corresponding to LETSLE, FEAM and VQNGL were separated and then detected within 15 min by the HPLC as shown in Fig. 2A. It means that the three N-terminal free peptides of LETSLE, FEAM and VQNGL were enzymatically produced at pH 5.5 from their acetyl substrates and successfully reacted with the catechol reagent in a buffered borate aqueous solution (pH 7.0) to generate corresponding FL compounds, which could be sensitively detected by the HPLC with the FL detector. Additionally, low background noises in the chromatogram indicate that most other components in the enzymatic reaction (e.g., buffers, proteins, nucleic acids, free amino acids and lipids) did not disturb the FL detection for the three peptide products.

The quantitative determination of the LETSLE, FEAM and VQNGL products was performed by the HPLC analysis using their standard curves (Fig. 2B). Areas of their FL peaks in the chromatogram were calculated and plotted against those of peptides. Proportional relationship between peptide concentrations and FL signals could be obtained over a wide concentration range (1–3000 pmol per injection) for each peptide (*R*^2^ = 0.97–0.99). Detection limits were 0.5–1.0 pmol per injection at a signal-to-noise ratio of 3. These results indicate that the present HFA can provide sensitive and simultaneous determination for peptides produced by the enzymatic reaction with HIV-PR.

### Activity assay of HIV-PR and its mutants

The wild-type HIV-PR gene was derived from the pNL4-3 HIV-1 clone and expressed in *E. coli* cells. We determined the Wt HIV-PR concentration in cell lysate with a quantitative immunoblotting method against a standard curve of a commercially available purified HIV-PR. The cell lysate was directly used and measured for the HIV-PR activity without further purification.

**Figure 3:**
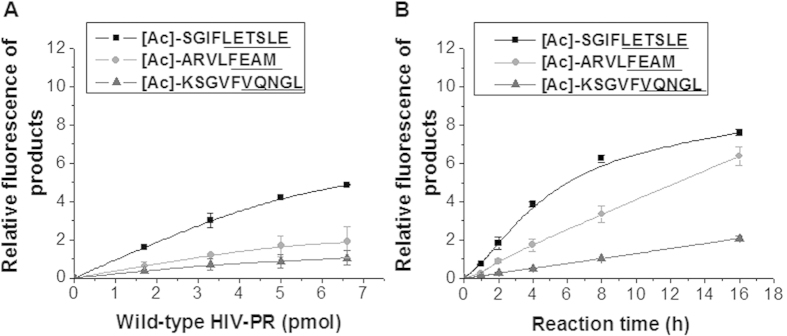
FL detection of HIV-PR activity in the cell lysate using a mixture of three acetyl substrates of 200 μM [Ac]-SGIFLETSLE, 200 μM [Ac]-ARVLFEAM and 800 μM [Ac]-KSGVFVQNGL, the enzyme reaction contained (**A**) different amounts (1.7–6.6 pmol) of HIV-PR (50 μl) at 37 °C for 4 h, and (**B**) 5 pmol of wild-type HIV-PR at 37 °C for different incubation times (1–16 h).

The cell lysates containing different concentrations of the wild-type HIV-PR were incubated with the three substrates for 4 h, followed by the FL reaction and HPLC analysis (Fig. 3A). Their FL peaks corresponding to the peptide products of LETSLE, FEAM and VQNGL were proportionally increased with the amounts (1.7–6.6 pmol) of the HIV-PR in the enzymatic reaction mixture. This result means that the present HFA can measure the proportional activity of HIV-PR depending on the enzyme concentration. The cell lysates that contained 5 pmol of the wild-type HIV-PR were incubated with the three acetyl substrates at 37 °C for prolonged periods (Fig. 3B). The HIV-PR activity was constant for at least 6 h, indicating that all three substrates were enzymatically cleaved by HIV-PR. Thus, the present assay can be used to measure specific HIV-PR activities in cell lysates.

**Figure 4:**
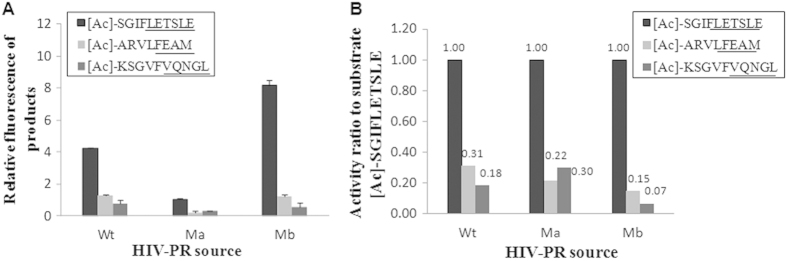
(**A**) FL production of the cleaved peptide fragments from three acetyl substrates by wild-type HIV-PR (Wt), mutant a (Ma) and mutant b (Mb); and (**B**) activity ratios of their peptide productions were normalized to the enzymatic production of LETSLE. The enzymatic reaction was performed at 37 °C for 4 h using 200 μM [Ac]-SGIFLETSLE, 200 μM [Ac]-ARVLFEAM and 800 μM [Ac]-KSGVFVQNGL. The LETSLE production from [Ac]-SGIFLETSLE was considered to be 1.00.

The present assay format could differentiate wild-type (Wt) HIV-PR and its mutants. Mutants, Ma and Mb were from drug-binding sites of G48V and V32I, respectively^24^. The cell lysate of Ma or Mb was incubated with three substrates under the same enzymatic conditions as for Wt HIV-PR, followed by the FL reaction and HPLC analysis. Enzymatically produced peptide fragments of LETSLE, FEAM and VQNGL from each substrate were determined, and their enzymatic productions differed slightly between Wt HIV-PR and its mutants (Ma and Mb) as shown in Fig. 4A. Therefore, the activities of Wt HIV-PR and its mutants were individually calculated for the three substrates. Figure 4B shows their activity ratios against the substrate of [Ac]-SGIFLETSLE for each enzyme source. The results explain that each activity ratio for the three substrates reflects different patterns for the mutants, and suggest that HIV-PR affinity varies by substrate with a different Km value.

### Evaluation of drug resistance

**Figure 5:**
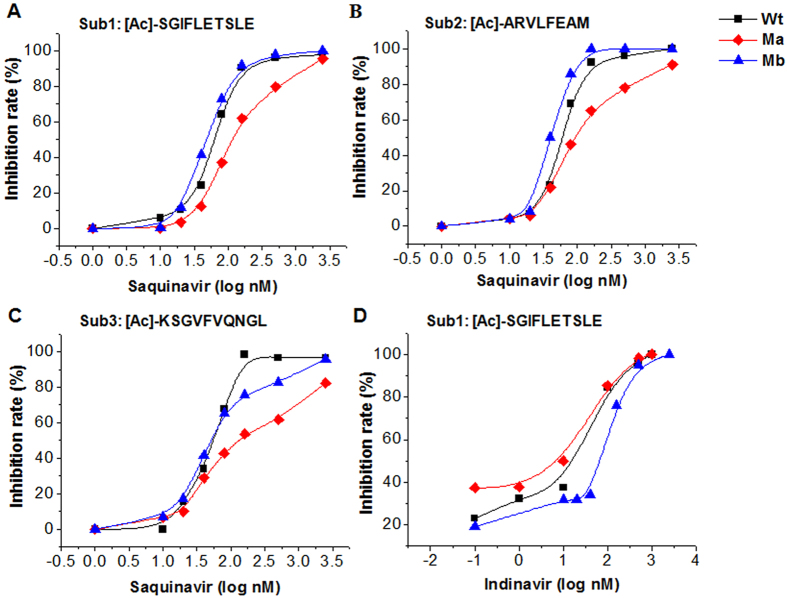
Inhibition curves for HIV-PR activities of Wt (■), Ma (♦) and Mb (▲) treated with different concentrations of saquinavir or indinavir. (**A**), (**B**) and (**C**) were evaluated with saquinavir as the protease inhibitor for each substrate of 200 μM [Ac]-SGIFLETSLE, 200 μM [Ac]-ARVLFEAM and 800 μM [Ac]-KSGVFVQNGL in the enzymatic reaction mixture; (**D**) was evaluated with indinavir for 200 μM [Ac]-SGIFLETSLE. Cell lysates containing HIV-PR or its mutants (5 pmol each in 50-μl enzymatic reaction mixture) were incubated with the three substrates and different concentrations of each inhibitor at 37 °C for 4 h, followed by the FL reaction and HPLC analysis.

The proposed assay format was further used to analyze the phenotypic drug resistance of the HIV-PR mutants for HIV-PR inhibitors of saquinavir, indinavir, lopinavir and ritovavir, which are clinically used. The HIV-PR mutants of Ma (G48V) and Mb (V32I) are reportedly resistant to saquinavir^46^,^47^,^48^ and indinavir^48^,^49^, respectively. Thus, different levels of the drug resistance of the mutants and Wt HIV-PR were first studied by using saquinavir and indinavir as HIV-PR inhibitors. In this experiment, the inhibition rates by these drugs on HIV-PR activity were measured by the decrease in the FL peak area corresponding to enzymatic peptide products. Figure 5A–C show the inhibition rate (%) of the enzyme activity for the substrates, [Ac]-SGIFLETSLE, [Ac]-ARVLFEAM and [Ac]-KSGVFVQNGL, respectively, depending on the saquinavir concentration in the enzymatic reaction mixture. As the results, the Ma mutant showed hysteretic inhibition curves with saquinavir for all the substrates, but not for indinavir. However, mutant Mb showed a hysteretic inhibition curve only with indinavir for the [Ac]-SGIFLETSLE substrate (Fig. 5D).

**Figure 6:**
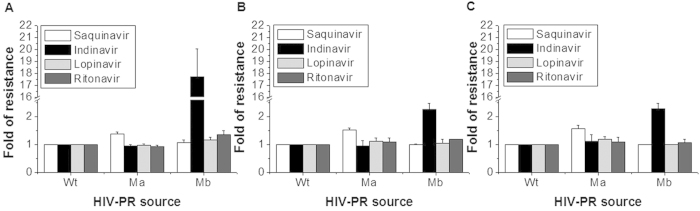
Drug resistance profiles for HIV-PR mutants evaluated by the present HFA method using the three substrates of (**A**) [Ac]-SGIFLETSLE, (**B**) [Ac]-ARVLFEAM and (**C**) [Ac]-KSGVFVQNGL. Cell lysates containing Wt, Ma or Mb HIV-PR were incubated with the three substrates at 37 °C for 4 h in the presence of 56 nM saquinavir, 4.5 nM indinavir, 11 nM lopinavir or 31 nM ritonavir. Each FL signal corresponding to the enzymatic products was used in the following formula to evaluate the fold resistance to each inhibitor drug: Fold resistance = 
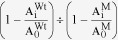
; In this formula, A_i_

To more readily assess the drug-resistance profile of each HIV-PR mutant, we determined each fold resistance value that was corresponded to the ratio of the inhibition rate at a single concentration of an inhibitor for each mutant (Ma and Mb) against that for Wt HIV-PR. Figure 6 depict the drug resistance profiles for the HIV-PR mutants. In this experiment, we used four inhibitor drugs (saquinavir, indinavir, lopinavir and ritonavir) towards the three substrates and calculated each value by the formula shown in the caption of Fig. 6. The results explain that Ma showed weak resistance to saquinavir, and not to other drugs; whereas Mb showed strong resistance to indinavir; however, neither Ma nor Mb were resistant to lopinavir or ritonavir.

The above data for IC_50_ doses and fold change in HIV-PR inhibition especially with sauinavir and indinavir were listed in Table 1. The data represent that the mutants, Ma and Mb clearly gave higher IC_50_ and fold change values with sauinavir and indinavir, respectively than Wt HIV-PR. It means that Ma is resistant to saquinavir, while Mb is resistant to indinavir.

## Discussion

By taking advantage of the simplicity and sensitivity of the FL reaction of *N*-terminal free oligopeptides with catechol^33^, we have developed a novel method for phenotypic differentiation of drug-resistant HIV-PR mutants. Using this method, we obtained IC_50_ values of several HIV-PR inhibitors by the simultaneous detection of three FL peaks that corresponded to enzymatically produced peptides from three substrates, and thus could evaluate drug resistance of two representative mutants (Ma and Mb) of HIV-PR according to the IC_50_ doses of inhibitor drugs (Fig. 5). We determined the fold changes for the drug resistance profiles using several HIV-PR inhibitors towards the three substrates for the HIV-PR mutants (Fig. 6). The values of the IC_50_ dose and fold change of two inhibitors, saquinavir and indinavir that are clinically used, were also listed in Table 1. Fold changes in resistance evaluation could help quickly produce extensive drug-resistance profiles of HIV-PR in patients. Those results were consistent with their reported phenotypes^48^, which support that the present HFA method can allow the phenotypic assay of drug-resistant HIV-PR correctly, although more mutant samples are needed to determine cutoff values for different drugs.

The present HFA method uses three different substrates simultaneously, and thus makes the outcome more informative and reliable. Resistance mutations in HIV-PR reduce the binding affinity of PR to its inhibitors and other substrates to varying degrees^14^, so using more than one substrate can avoid the possible influence of substrate preference of HIV-PR mutants. The HFA method could be performed within 1–2 weeks, which is much quicker than clinically used recombinant virus-based methods that need 3–4 weeks^30^. Turnaround time for HFA would be further decreased by using advanced HPLC that allows automatic sampling, automatic measurement and high-throughput analysis. The present HFA permitted direct use of cell lysate and highly sensitive detection of enzymatic peptide products. It suggests that the method might directly use specimens such as patients’ blood and lymph fluid. Further studies with patient’s blood specimens should be needed for the practical application of HFA, though we could not get AIDS patient’s specimens.

In principle, development of similar assays for phenotypic differentiation of drug-resistant HIV-reverse transcriptase, and a combination assay for both drug-resistant HIV-PR and HIV-reverse transcriptase is possible. Taken together, the present HFA method shows great potential to enable inexpensive, rapid, informative and reliable phenotypic differentiation of drug-resistant HIV-PR mutants.

## Methods

### Preparation of peptide substrates and their fragment peptides

Three acetyl HIV-PR substrates of acetyl-Ser-Gly-Ile-Phe-Leu-Glu-Thr-Ser-Leu-Glu ([Ac]-SGIFLETSLE), acetyl-Ala-Arg-Val-Leu- Phe-Glu-Ala-Met ([Ac]-ARVLFEAM) and acetyl-Lys-Ser-Gly-Val-Phe-Val-Gln-Asn-Gly-Leu ([Ac]-KSGVFVQNGL), and their fragment peptides of LETSLE, FEAM and VQNGL were purchased from Sigma-Genosys Japan (Tokyo, Japan). These peptides were dissolved in water and stored at −20 °C.

### Preparation of Wt HIV-PR and its drug-resistant mutants

A Wt HIV protease gene (Gene Bank accession no. M19921) was cloned into the pMAL-c2x plasmid (New England Biolabs, Ipswich, UK) to make a Wt HIV-PR-expressing vector (pMAL-c2x-PR) using conventional cloning techniques and primers: 5′-GAGGGAAGGATTTCATCCTTCAGCTTCCCTCAGATCACTCTTTGG-3′ and 5′-AGGAAGCTTTTAAAAATTTAAAGTGCAGCC-3′. As two drug-resistant mutations (G48V^47^ and V32I^48^) for the HIV-1 protease gene were reported, their genes were introduced into the pMAL-c2x-PR to generate two HIV-PR mutants, the Ma- and Mb-expressing vectors, respectively. The mutations were carried using a QuickChange II Site-Directed Mutagenesis kit (Agilent Technologies, CA, USA) and primers: 5′-GATGGAAACCAAAAATGATAGTGGGAATTGGA GG-3′ and 5′-CAGGAGCAGATGATACA ATTTTAGAAGAAATGAATTTGCCAGG-3′. The vectors were confirmed by DNA sequencing. Each vector containing the Wt or mutant HIV-PR gene was transformed into E. coli strain DH5α cells. The transformed E. coli cells were cultured in LB broth until they attained OD_600_ of 0.6–0.8; 1.0 mM ITPG was then added to induce HIV-PR expression over 2 h. Cells were centrifuged at 13,400 × g for 3 min at 4 °C and then washed with 1 × PBS. After re-suspension in water, the cells were sonicated three times in ice water at power 40 with 10-sec intervals (Model 300 V/T, BioLogics Inc. NC, USA). The lysate was centrifuged at 13,400 × g for 3 min at 4 °C; its supernatant was stored at −40 °C. The HIV-PR concentration in the lysate was determined by western blotting^50^ with mouse anti-HIV-PR antibody (Clone 1696, ExBio, Praha, Czech Republic) and HRP-conjugated anti-mouse IgG antibody (Nacalai, Tokyo, Japan). Commercial HIV-PR (Abcam, Cambridge, England) was used for quantitative calibrations.

### Enzymatic reaction

The lysate that contained 5 pmol of HIV-PR or HIV-PR mutant was directly used for the enzymatic reaction (50 μl), which was performed at 37 °C for 4 h in 50 mM sodium acetate buffer (pH 5.5) containing 200 μM [Ac]-SGIFLETSLE, 200 μM [Ac]-ARVLFEAM and 800 μM [Ac]-KSGVFVQNGL as multi-substrates. After the enzymatic reaction, the reaction mixture was subjected to the FL reaction followed by HPLC analysis. The enzymatically produced peptides from the substrates were simultaneously detected and their concentrations were analyzed by HPLC.

### FL reaction

Conditions for the FL reaction of peptides were as previously reported^33^,^39^, but reaction conditions for the enzymatically produced peptides were slightly modified as follows: a portion (50 μl) of the enzyme reaction mixture or an aqueous solution of synthetic standard peptide was successively mixed with 2.5 μl of 0.1 N NaOH, 10 μl of PBS, 25 μl of 0.3 M H_3_BO_3_–NaOH (pH 7.0), 50 μl of 2.5 mM catechol and 25 μl of 2 mM NaIO_4_. The mixture (162.5 μl) was then heated to 100 °C for 10 min. After the reaction, the mixture was immediately cooled in ice water to stabilize the FL compounds. Finally, the mixture was centrifuged at 13,400 × g for 3 min at 4 °C and the supernatant containing the FL compounds was subjected to HPLC.

### HPLC analysis

The FL compound quantities were analyzed by an HPLC system, which consisted of a pump with degasser (PU-2089 plus, JASCO, Japan), UV detector (UV-2070, JASCO), FL detector (FP-920, JASCO) and chart recorder (Ross, USA). The analytical column was TSK gel ODS-80Ts (Tosoh, Japan). The FL reaction mixture (5–20 μl) was injected into the HPLC system. Gradient elution of the mobile phase composed of eluent A (methanol), B (0.25 M H_3_BO_3_–NaOH, pH 7.0) and C (H_2_O) was performed for the separation as follows: 0–35% of A, 5% of B, and 95–60% of C for 0–25 min; 35–80% of A, 5% of B, and 60–15% of C for 25–26 min; 80% of A, 5% of B, and 15% of C for 26–33 min; 80–0% of A, 5% of B, and 15–95% of C for 33–34 min; followed by at least 10 min for the reconstitution of the column before the next analysis. The flow rate was 1.0 ml/min. The FL detector was set at 400 nm for excitation and 490 nm for emission. The enzymatically produced peptides were quantified by measuring their FL peak areas in the chromatogram.

The inhibition rate of an inhibitor on HIV-PR activity was calculated by the relative decrease in product peptides. The concentration of the inhibitor that inhibited 50% the HIV-PR activity (IC_50_) on each substrate was calculated with the Forecast function of Excel software.

### Evaluation of drug resistance

Cell lysates containing HIV-PR (<5 pmol) were incubated with the three substrates as described above, with varying concentrations (0–2.5 μM) of therapeutic HIV-PR-inhibitors: saquinavir mesylate (Sigma, China), indinavir sulfate salt hydrate (Sigma, China), lopinavir (Santa Cruz Biotechnology, CA, USA) or ritonavir (Toronto Research Chemicals Inc., Canada). The enzymatically formed peptides were analyzed as described above.

## Author Contributions

M.K., Q.Z. and T.K. conceived and planned the study. Q.Z. and Z.Y. performed experiments and analyzed the data. M.K. and Q.Z. wrote the manuscript. All of the authors discussed the results and commented on the paper.

## Additional Information

**How to cite this article**: Zhu, Q. *et al.* Fluorometric assay for phenotypic differentiation of drug-resistant HIV mutants. *Sci. Rep.* **5**, 10323; doi: 10.1038/srep10323 (2015).

## Figures and Tables

is the FL peak area of each enzymatic product in the presence of each drug, and 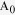 is the peak area of the product in the absence of the drug. Wt is wild-type HIV-PR, and M is its mutant.

**Table 1:** IC_50_ doses and fold changes in HIV-PR inhibition with saquinavir and indinavir.

**HIV-PR**		**Saquinavir**						**Indinavir**					
	**IC**_**50**_ **(nM)**			**Fold change**			**IC**_**50**_ **(nM)**			**Fold change**			
**Sub1**	**Sub2**	**Sub3**	**Sub1**	**Sub2**	**Sub3**	**Sub1**	**Sub2**	**Sub3**	**Sub1**	**Sub2**	**Sub3**	
Wt	56.7 ± 5.3	61.8 ± 1.7	56.7 ± 1.9	1.0	1.0	1.0	4.8 ± 1.9	4.5 ± 2.5	4.1 ± 1.6	1.0	1.0	1.0
Ma (G48V)	107.1 ± 18.3	108.9 ± 5.8	153.1 ± 28.7	1.9	1.8	2.7	2.7 ± 1.5	4.1 ± 0.5	3.3 ± 0.6	0.6	0.9	0.8
Mb (V32I)	56.2 ± 4.1	45.6 ± 4.6	55.2 ± 8.5	1.0	0.7	1.0	25.7 ± 2.0	22.7 ± 2.4	11.1 ± 1.9	5.3	5.0	2.7

IC_50_: inhibitor concentration to inhibit 50 percent of HIV-1 PR activity, displaying the mean ± SD of three independent experiments. Fold change: ratio of IC_50_ values between a mutant and wild-type HIV-1 PR based on the same substrate; Sub1: [Ac]-SGIFLETSLE; Sub2: [Ac]-ARVLFEAM; Sub3: [Ac]-KSGVFVQNGL.
